# ﻿Taxonomic studies on the genus *Hydrocotyle* (Apiales) from China: The distribution and morphology of *H.chiangdaoensis*, with *H.calcicola* reduced to a synonym

**DOI:** 10.3897/phytokeys.255.149589

**Published:** 2025-04-30

**Authors:** Jun Wen, Jun-Wen Zhu, Ren-Bin Zhu, Chun-Feng Song

**Affiliations:** 1 Jiangsu Key Laboratory for the Research and Utilization of Plant Resources, Institute of Botany, Jiangsu Province and Chinese Academy of Sciences (Nanjing Botanical Garden Mem. Sun Yat-Sen), Nanjing, China Institute of Botany, Jiangsu Province and Chinese Academy of Sciences (Nanjing Botanical Garden Mem. Sun Yat-Sen) Nanjing China; 2 Xishuangbanna Tropical Botanical Garden, Chinese Academy of Sciences, Mengla, Yunnan, China Xishuangbanna Tropical Botanical Garden, Chinese Academy of Sciences Mengla China

**Keywords:** *Hydrocotyle*, synonym, taxonomy, Yunnan

## Abstract

Based on observations of living plants of *Hydrocotylecalcicola* in the field, together with examination of herbarium specimens and descriptions of both *H.calcicola* and *H.chiangdaoensis* (including type material), we demonstrated that *H.calcicola* is a synonym of *H.chiangdaoensis*. The species was previously compared with *H.sibthorpioides*; our phylogenetic analysis revealed that *H.chiangdaoensis* and *H.sibthorpioides* belong to different lineages, the former being closely related to the larger-leaved clade.

## ﻿Introduction

The genus *Hydrocotyle* Tourn. ex L. contains approximately 180 species (Plants of the World Online, http://www.plantsoftheworldonline.org/). The native range of this genus is cosmopolitan, with Australia, South America, and China as three diversity distribution centers. *Hydrocotyle* was formerly a member of the family Apiaceae, but was later transferred to Araliaceae based on molecular phylogenetic studies (Chandler and Plunkett 2004; Plunkett et al. 2004; Nicolas and Plunkett 2009). Subsequently, Wen et al. (2024) highlighted the independent status of this genus within Apiales based on the analysis of chloroplast genome data. All these analyses have suggested that the genus *Hydrocotyle* is key to further study on the evolution of Apiales.

We have conducted a comprehensive study of *Hydrocotyle* in China and found that there are 16 species, 2 varieties, and 2 subspecies in the country, among which Yunnan Province is most species-rich (Sheh et al. 2005; Pimenov 2017). Some species occurring in Yunnan were also found in adjacent areas, such as *H.sibthorpioides* Lam., *H.javanica* Thunb., and *H.siamica* Craib, which were also found in Myanmar and Thailand (Watson and Smith 2004; Pimenov 2017). Studies on Myanmar’s plant diversity have been conducted in recent years. A newly recorded species of *Hydrocotyle*, *H.chiangdaoensis* Murata, has been reported by Kang et al. (2018), which was formerly known to be endemic to Thailand. However, the photo of this species was very similar to *H.calcicola* Y.H.Li, which was endemic to Xishuangbanna, Yunnan Province, China (Sheh et al. 2005). Based on this, we conducted a comparative study between the two species.

**Figure 1.**
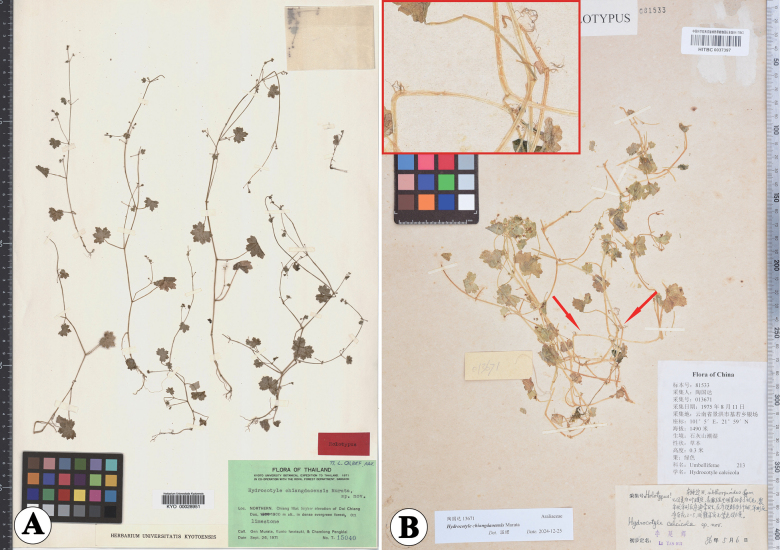
Holotype sheets of *Hydrocotylechiangdaoensis* (**A**) and *H.calcicola* (**B**). The arrows indicate the roots of the species.

**Figure 2.**
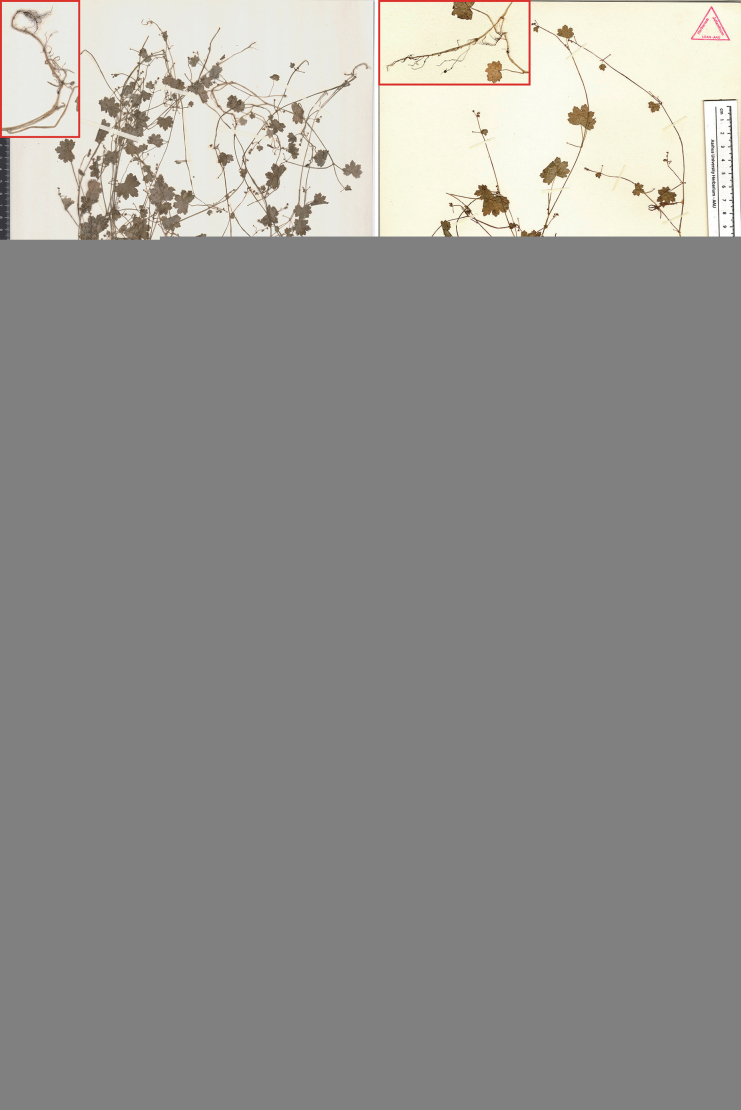
Isotype sheets of *Hydrocotylechiangdaoensis*. The arrows indicate the roots of the species.

*Hydrocotylechiangdaoensis* was recognized as a new species by G. Murata in 1973 and was described based on *Murata G. et al.* T-15040 (holotype KYO 00028951, Fig. 1A; isotypes KYO 00028952, AUU, TI 00083127, and L 0008361, Fig. 2A–D), collected in Doi Chiang Dao, Northern Chiang Mai, Thailand (Murata 1973). In the protologue, Murata emphasized four distinguishing features of *H.chiangdaoensis* – palmate-dissected stipules, annual habit, not emitting any roots from the nodes, and limestone area endemic. Specifically, the annual habit was speculated because the stem and branches have terminal inflorescences (Murata 1973).

A collection of this species from Doi Chiang Dao (*M. Tagawa & K. Iwatsuki* T-4389, L.2583137) was reported by Hiroe as *H.siamensis* H. Wolff (1921) (Hiroe 1967). The latter name is a synonym of *H.siamica* Craib (Craib 1911), based on the same gatherings (*Hosseus* no. 192; E00000016, P00757659, L.2595136, and M0168575). *Hydrocotylesiamica* was described with long peduncled umbels (*Umbellae* axillarea, solitariae, pedunculo ad 10.5 cm. longo brunneo-puberulo suffultae) and large leaves (*Folia* pentagona, vix vel fere ad medium lobata, ad 9 cm. longa et 10 cm). The diagnostic characters of the specimen L.2583137 (subsequently identified as *H.chiangdaoensis*) are quite different from *H.siamica*, with short peduncled umbels and small leaves.

The oldest specimen of this species we found was collected by Kerr in 1922 (*Kerr* 6530, K005513556) from Doi Chiang Dao in Northern Chiang Mai, Thailand. For nearly 100 years since then, this species has only been seen in Doi Chiang Dao. Kang *et al.* (2018) made a new distribution record of this species in Ywangan Township, Southern Shan State, Myanmar, when they conducted joint floristic surveys of this area in 2017. Since then, it has not been seen anywhere else.

**Figure 3.**
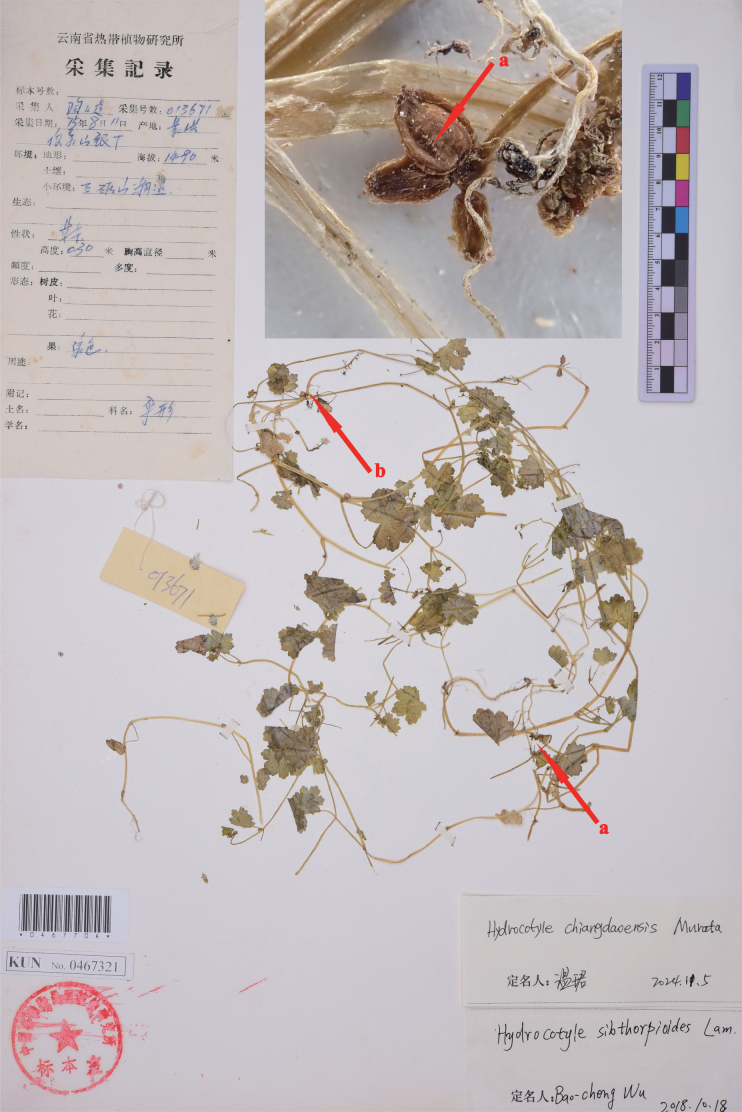
Isotype sheet of *Hydrocotylecalcicola*. The arrows indicate fruit (a) and root (b) of the species.

*Hydrocotylecalcicola* Y.H.Li was described on the basis of *G.D.Tao* 13671 (holotype HITBC0037397, Fig. 1B; and isotype KUN0467704, Fig. 3) from Jiluo Shan (according to textual research, it should be Jinuo Mountain), Jinghong City, Yunnan, China (Li and Zhang 1989). In the protologue, Li and Zhang highlighted the features that set this species apart from *H.sibthorpioides* Lam.: membranous leaves conspicuously covered with sparse spinous hairs, umbels of cymes usually terminal with opposite leaves, short and slender peduncles, umbels of 2–5 flowers, and mature fruits not purplish-spotted. Liou (1997) treated *H.calcicola* as a variety of *H.sibthorpioides* due to the weak difference only focused on flowers and fruit. This treatment has not been accepted by *Flora of China* (Sheh et al. 2005), because the habitat of *H.calcicola* was very special. It was noted in the protologue that the habitat of *H.calcicola* was also limestone, and the stipules were irregular, i.e. very similar to *H.chiangdaoensis*. Upon comparing the original descriptions of the two species, we found that many features of both were similar, with only minor differences in the descriptions of their life forms, roots, and fruit. *Hydrocotylechiangdaoensis* was described as “not emitting any roots from the nodes”, with “annual habit” and “fruit broadly ovate, papillose-setulous, truncate or subcordate at the base”; but *H.calcicola* was described as “perennial herbs”, “rooting at nodes”, and “fruits subcordate, smooth on the outside, ribs conspicuously convex”. However, the annual habit of *H.chiangdaoensis* was speculated on the basis that the stem and branches have terminal inflorescences, which were also a feature of *H.calcicola* (Murata 1973; Li and Zhang 1989). We suggested that *H.calcicola* might be a synonym for *H.chiangdaoensis* based on their similar traits and the order of publication years.

This study aimed to determine the identity of *H.calcicola* and elucidate the distribution and morphological variation of *H.chiangdaoensis*. Our research was conducted mainly on observations of herbarium specimens (including the type material) and living plants in the field. The phylogenetic tree was reconstructed to determine the phylogenetic placement of *H.chiangdaoensis*.

## ﻿Materials and methods

### ﻿Morphological analysis

For morphological comparisons, we thoroughly examined specimens or high-resolution images of related *Hydrocotyle* Tourn. ex L. from the following herbaria: AAU, ANUB, AU, BC, BJM, BKF, BM, CAL, CDBI, CSFI, CSH, CZH, E, FJSI, G, GFS, GH, GNUG, GXMG, GZTM, HGAS, HITBC, HNWP, HZ, IBK, IBSC, IMC, IMDY, JIU, JJF, K, KUN, KYO, L, LBG, LE, M, MA, MPU, MW, NAS, NY, P, PE, PEY, QNUN, SANU, SM, SYS, SZ, SZG, TAI, TI, TNS, US, WCSBG, WU, WUK, XBGH, XZ, ZY. Two populations of *H.calcicola* were observed in the field, one from the type locality (Jinuo Mountain, Jinghong City, Yunnan) and another from Ning’er County, Pu’er City, Yunnan. A comprehensive analysis of old herbarium specimens and fresh materials collected during our fieldwork was presented as the result of morphological comparisons.

### ﻿Phylogenetic analysis

The complete chloroplast genomes were used to reconstruct the phylogenetic trees of the genus *Hydrocotyle* according to the analysis of Wen et al. (2024). A sample of *H.calcicola* from Pu’er was newly sequenced using the Illumina Novaseq 6000 platform at Novogene (Beijing, China), with paired-end reads 2 × 150 bp. The chloroplast genome was assembled using NOVOPlasty v4.3.3 (Dierckxsens et al. 2017). The assembled sequence was checked and annotated under Geneious Prime 2023.2.1 (created by the Biomatters development team, Ltd.), and was subsequently uploaded to the National Center for Biotechnology Information (NCBI) with accession number PV094900.

A total of twelve taxa from the genus *Hydrocotyle* have been sampled for phylogenetic analysis, including nine species. Two species from Apiaceae were selected as outgroups [*Dickinsiahydrocotyloides* Franch., and *Eryngiumcampestre* L.]. The whole genome sequence matrix was generated from MAFFT v7 (Katoh and Standley 2013). Two methods were employed to conduct phylogenetic analysis: Bayesian inference (BI) and maximum likelihood (ML). The best-fit model “GTR + I + G” was recommended by jModelTest v2.1.4 (Darriba et al. 2012). The ML analysis was performed by RAxML v8.2.4 (Stamatakis 2014). Rapid bootstrap analysis was implemented using 1000 bootstrap replicates to search for the best ML tree. MrBayes v3.2.7a (Ronquist et al. 2012) was employed to conduct the BI analysis. Two independent Markov chain Monte Carlo (MCMC) runs were performed, each with three heated chains and one cold chain for 10,000,000 generations. The average standard deviation of split frequencies should approach zero. Each run started with a random tree, sampling trees every 1000 generations, with the initial 25% discarded as burn-in. The posterior probability (PP) and bootstrap support (BS) were used to measure the supports of the phylogenetic tree implemented under BI and ML, respectively. The final trees were viewed and edited in FigTree v1.4 (Rambaut 2012).

## ﻿Results and discussion

The type materials of *Hydrocotylechiangdaoensis* (Figs 1A, 2) are small erect herbs, leaves cordate-orbiculate, gradually smaller above, slightly hairy above towards the veins, glabrous beneath, palmately 7-lobed, lobes usually 3-toothed, teeth obtuse, blades (0.6)1–2(2.5) cm long, (0.7)1.5–2.5(3) cm wide; petioles (0.7)1.5–3.5(5) cm long; stipules membranous flabellate-orbiculate, 1–5 mm wide, palmately dissected; umbels terminal or axillary of the branch or opposite the leaves of the branches, terminal cymose umbels 2–3, thin peduncles 0.5–6 mm long, umbel 2–4 flowers, pedicels very short, membranous bracts minute ovate-lanceolate; petals ovate-lanceolate; fruit broadly ovate, 1–1.2 mm long, papillose-setulous, base truncate or subcordate, stylopodia shortly conoid, short styles reflexed at the end. The root system of this species was described as “not emitting any roots from the nodes” in the protologue. However, we found that some individuals have roots growing from two or three nodes at the base (Fig. 2A–C).

**Figure 4.**
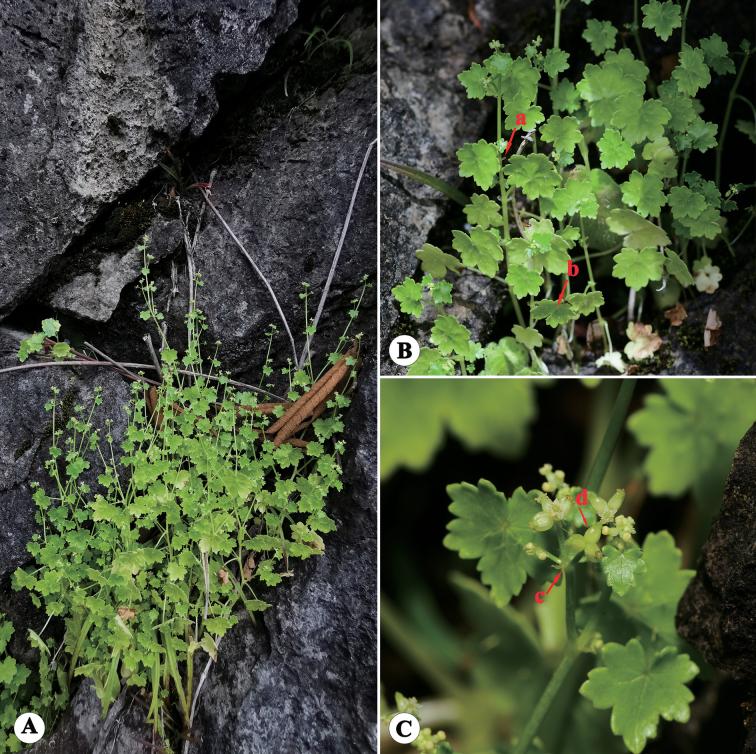
*Hydrocotylechiangdaoensis* in the wild (China, Yunnan, Jinghong, Mountain Jinuo, the type locality of *H.calcicola*) **A** habitat **B** habit, with the arrows indicating stipule (a) and leaf epidermal spines (b) **C** the terminal inflorescence, with the arrows indicating cymose umbels (c) and fruit (d). Photographed by Ren-Bin Zhu.

**Figure 5.**
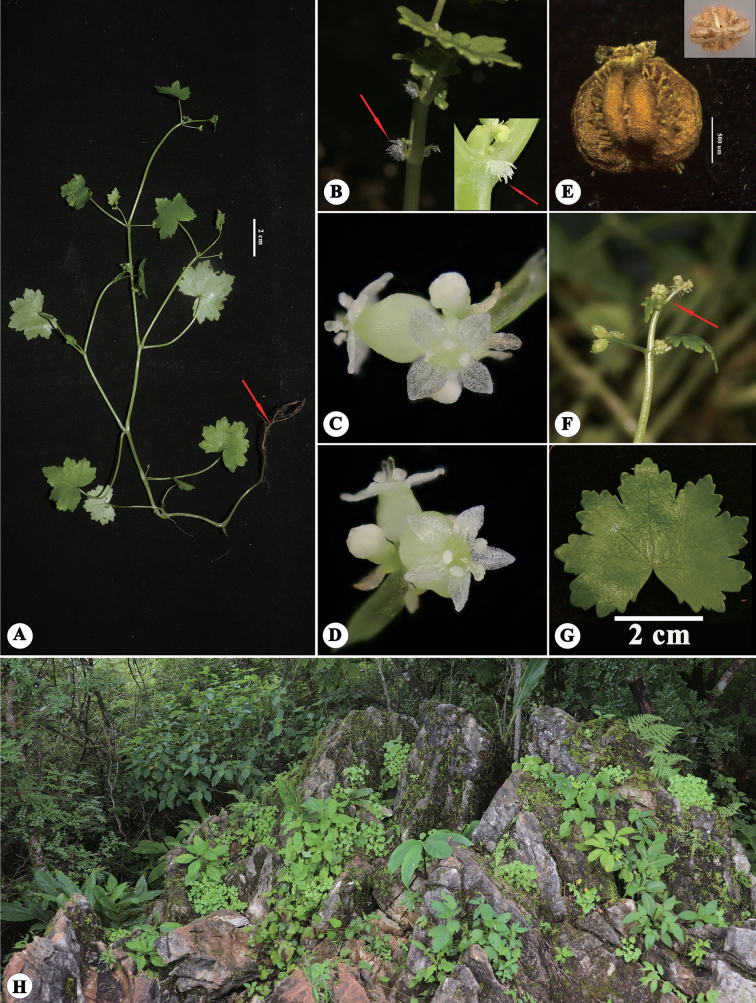
*Hydrocotylechiangdaoensis* in the wild (China, Yunnan, Pu’er) **A** plant, with the arrow indicating root **B** stipules **C, D** flowers **E** fruit **F** the terminal inflorescence **G** leaf **H** habitat. Photographed by Jun Wen.

Type specimens of *H.calcicola* (Figs 1B, 3) have shown that this species was rooting only at nodes of the basal stem and never elsewhere, the same as *H.chiangdaoensis*. The holotype of *H.calcicola* has membranous leaves subrounded or cordate, conspicuously covered with sparse spiny hairs above, glabrous beneath, cordate at the base, 5–7-lobed at the apex, lobes broadly obovate, crenate at the margin, 6–8 palmate nerves, 0.5–1.5 cm long, 0.7–2.5 cm wide; petiole 0.7–3 cm long, glabrous; stipules small, kidney-shaped, thinly branched, irregularly; cymose umbels usually terminal with opposite leaves, umbels 2–3, slender peduncles not quite equilong, laterals about 1 cm long, middle 1–2 mm long, umbel 2–5 flowers, flowers sessile, bracts ovate-lanceolate, about 1 mm long, membranous; petal ovate, about 0.5 mm long, white, filaments equal to or slightly shorter than the petal, anthers ovate; style about 0.2 mm long; fruits subcordate, 1–1.3 mm long, 0.8–1.2 mm wide, flat on both sides, smooth or with papillose setae on the outside, ribs conspicuously convex (Fig. 1B). Fruits with papillose setae were also discovered in the isotype (Fig. 3). These morphological features were corroborated through field observations of living plants in Jinuo Mountain, Jinghong City, Yunnan (Fig. 4), the type locality of *H.calcicola*. At the same time, after observing live plants in the type locality, we found that the young fruits are sometimes smooth, but most fruits have papillose setae in the furrow. The additional observations of living plants in Pu’er show that most of the traits of this population were consistent with the description of *H.calcicola*, including habitat, roots, leaves, stipules, terminal umbels, and fruits (Fig. 5). Our analysis added some additional information about this species. Two types of stipules in this population were observed, one irregularly divided and one palmately dissected (Fig. 5B). The latter form is the same as that of *H.chiangdaoensis*. Two petal numbers (4 and 5) have been observed in this population, marking the first time such numbers have been reported in *Hydrocotyle*.

In our examination of the type specimens (Figs 1B, 3) and field observation (Figs 4, 5) of *H.calcicola*, particularly concerning critical diagnostic characters, such as roots, leaves, stipules, inflorescence, and fruits, no significant differences were observed when compared to *H.chiangdaoensis*. In conclusion, *H.calcicola* is morphologically indistinguishable from *H.chiangdaoensis*. Therefore, we propose to synonymise *H.calcicola* with *H.chiangdaoensis*.

**Figure 6.**
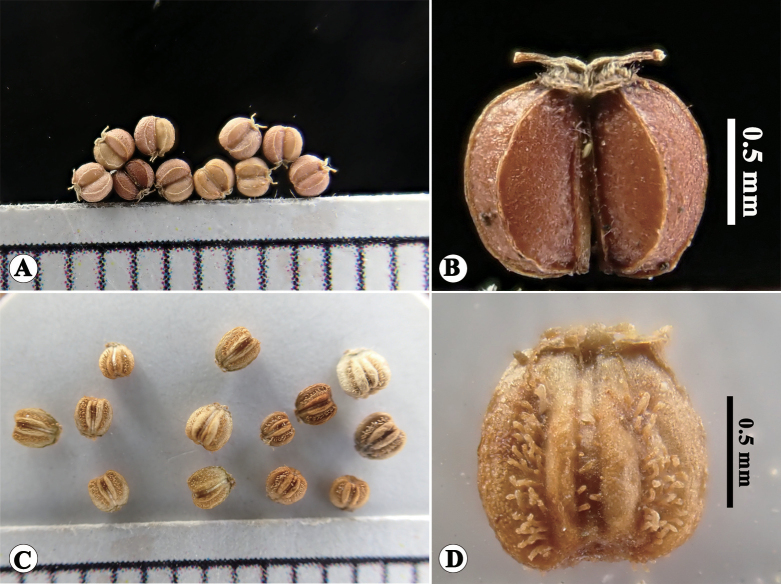
Fruits of *Hydrocotylesibthorpioides* (China, Hunan, Zhangjiajie, NAS00638796) and *H.chiangdaoensis* (China, Yunnan, Pu’er) **A, B** fruits of *H.sibthorpioides***C, D** fruits of *H.chiangdaoensis*.

**Figure 7.**
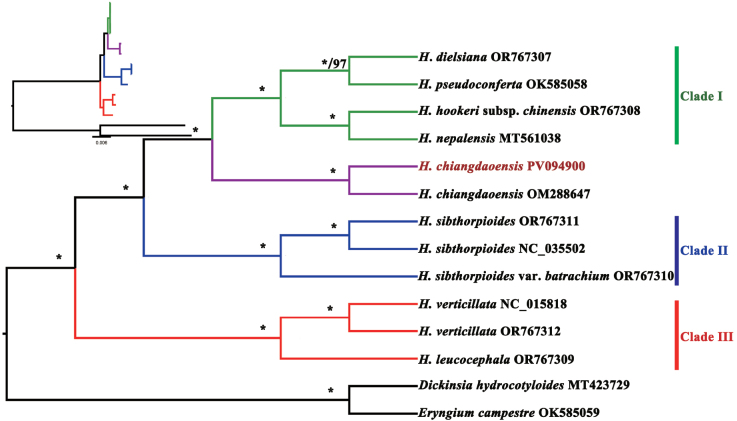
Phylogenetic tree inferred from 14 whole chloroplast genome sequences. Support values marked above the branches follow the order Bayesian inference (PP, posterior probability) / maximum likelihood (BS, bootstrap support), * represent the best support (100%).

As previously noted, *H.calcicola* was compared with *H.sibthorpioides* by its discoverers (Li and Zhang 1989) and subsequently treated as a variety of the latter (Liou 1997). When we examined the specimens, we also found that the specimens of *H.calcicola* were often misidentified as *H.sibthorpioides* by various researchers. Thus, we here provide a detailed morphological comparison between the two species in Table 1, Fig. 6. The results show that there are clear morphological distinctions (especially in roots, inflorescences, and mericarps) between the two species. The phylogenetic analysis based on the chloroplast genome was implemented to clarify the relationship between these two species. Phylogenetic trees reconstructed by BI and ML methods both recovered a stable topology within the genus *Hydrocotyle* with strong support. Within the genus, *H.chiangdaoensis* and *H.sibthorpioides* were located in two different branches (Fig. 7). Two accessions of *H.chiangdaoensis* were gathered together, forming a sister branch of the larger-leaved clade (Clade I, Wen et al. 2024). The results of the morphological and phylogenetic analyses confirm that *H.chiangdaoensis* and *H.sibthorpioides* are different taxonomic entities.

**Table 1.** Morphological comparisons between *Hydrocotylesibthorpioides* and *H.chiangdaoensis*.

	*H.chiangdaoensis*	*H.sibthorpioides*
Habitats	limestone areas, shady and moist places	forests, wet grassy places, stream banks
Roots	rooting only at nodes of the basal stem and never elsewhere	rooting at the nodes
Stems	erect or ascending, slender, branched, with ascending branches 2–20 cm long	weak, slender, filiform, creeping, diffusely branched
leaves	membranous leaves subrounded or cordate, palmately 5–9-lobed, conspicuously covered with sparse spiny hairs above towards the veins, glabrous beneath	membranous, leaf blade reniform-rounded, base cordate, crenate, shallowly 5–7-lobed or nearly entire, variably hairy, adaxially glabrous and abaxially sparsely strigose along veins, or sometimes both surfaces glabrous or densely puberulous
Stipules	membranous, thin, palmate-dissected or irregularly dissected, without spots	membranous, entire or irregularly lobed, with purplish stains
Inflorescences	umbels usually solitary at the nodes, with terminal cymose umbels	umbel solitary at the nodes
Rays of umbels	terminal 2–3, axillary 1	1
Bracteoles	membranous, ovate-lanceolate, minute	ovate to ovate-lanceolate, membranous, with bright yellow glands
Umbellules	2–5-flowered, sessile flowers	5–8-flowered
Flower	filaments equal to or slightly shorter than the petals, petals 4–5	filaments equal to or slightly longer than the petals, petals 5
Mericarps	broadly ovate or subcordate, papillose-setulous or sometimes smooth on the outside, ribs conspicuously convex; not easy to separate when mature	broadly globose, greenish-yellow when young, glabrous, covered with purplish stains when mature, intermediate ribs very prominent; easily separated when mature

### ﻿Taxonomic treatments

#### ﻿ Hydrocotyle chiangdaoensis

Taxon classificationPlantaeApialesAraliaceae

Murata, Acta Phytotax. Geobot. 25: 97. 1973.

E5FA26BE-BE53-55D9-8FCA-E412C44014EC

= Hydrocotylecalcicola Y.H.Li, Guihaia 9: 25. 1989. = Hydrocotylesibthorpioidesvar.calcicola (Y.H.Li) S.L.Liou, Fl. Yunnanica 7: 363. 1997. Type: China. Yunnan, Jinghong City, Jinuo Mountain, 11 August 1975, *G.D.Tao 13671* (holotype: HITBC0037397; isotype: KUN0467704).

##### Type.

Thailand. Northern Chiang Mai, 26 September 1971, *Murata G. et al.* T-15040 (holotype: KYO00028951; isotype: KYO00028952, L0008361; TI00083127; AAU). Figs 1A, 2.

##### Etymology.

This species is currently found only in limestone areas, so we have retained the Chinese name 石山天胡荽 [Pinyin: shí shān tiān hú suī] of *Hydrocotylecalcicola*.

##### Description.

Herbs, 15–60 cm tall, glabrous. Stems erect or ascending, slender, branched, with ascending branches 2–20 cm long, rooting only at nodes of the basal stem and never elsewhere. Membranous leaves subrounded or cordate, gradually smaller above, palmately 5–9-lobed, lobes usually crenate at the margin, conspicuously covered with sparse spiny hairs above towards the veins, glabrous beneath, 0.5–4.5 cm long, 0.7–4 cm wide. Petiole 0.7–3.5 (5) cm long, glabrous. Stipules membranous flabellate-orbiculate, 1–5 mm wide, palmate-dissected or irregularly. Terminal umbels cymose, umbels 2–3, and usually solitary at the other nodes; slender peduncles not quite equaling, 0.5–10 mm long; umbel 2–5-flowered, sessile; bracts ovate-lanceolate, about 1 mm long; petals ovate-lanceolate, about 0.5 mm long, white, 4 or 5; membranous filaments equal to or slightly shorter than the petals; anthers ovate; style about 0.2 mm long. Mericarps broadly ovate or subcordate, 1–1.3 mm long, 0.8–1.2 mm wide, with papillose setae in the furrow or sometimes smooth on the outside, ribs conspicuously convex. The mericarps are not easy to separate when mature.

##### Distribution.

China (Yunnan: Jinghong, Lincang, Pu’er), Myanmar (Southern Shan State: Ywangan Township), and Thailand (Chiang Mai).

##### Habitat.

The species grows on limestone at elevations of 1300–2175 m above sea level, always in dense evergreen forests, shady and moist places.

##### Phenology.

Flowering and fruiting from July to November.

##### Additional specimens examined.

China. **Yunnan Province** • Jinghong City, Jinuo Mountain, 21°59'N, 101°05'E, alt. 1490 m, 11 August 1975, *G.D.Tao 13671* (HITBC081533, KUN0467704) • Lincang City, Yongde County, 24°09'27.3"N, 99°14'58"E, alt. 1923 m, October 2015, *LiYL1395* (KUN1372015) • Pu’er City, Lancang County, 26 September 1993, *Y.Y.Qian* 3024 (HITBC0122528) • Pu’er City, Ning’er County, 23°04'12.74"N, 101°01'43.3"E, alt. 1733–1840 m, 23 August 2023, *WJ2361* (NAS00714697–NAS00714702) • Pu’er City, Ning’er County, 23°04'22.6"N, 101°01'37.7"E, alt. 1798 m, 28 September 2020, *D.P.Ye 1994* (HITBC0063922, PE02521420) • Pu’er City, Ximeng County to Lancang County, 22°45'N, 99°40'E, alt. 1900 m, 24 October 1989, *G.D.Tao et al.* 39828 (HITBC0122527, KUN0462683).

Myanmar. **Southern Shan State** • Ywangan Township, 21°13'50.2"N, 96°31'03.7"E, alt. 1372 m, 6 October 2017, *Kim et al. MM-6405* (HHU).

Thailand. **Chiang Mai** • Doi Chiang Dao, alt. 1300–1900 m, 27 September 1971, *G. Murata et al. T-15147* (K005513596, L.2583778, P03259185) • Doi Chiang Dao, alt. 1900–2175 m, 14 September 1967, T. *Shimizu et al. T-10125* (K005513595, L.2583136) • Doi Chiang Dao, alt. 2000 m, 16 July 1958, *Th. Sørensen et al. 4172* • Doi Chiang Dao, alt. ca. 1700 m, 3 November 1922, *A.F.G. Kerr 6530* (K005513556) • Doi Chiang Dao, 18 October 1926, *no. 412* (K005513592) • Doi Chiang Dao, alt. 1400–1800 m, 5 January 1966, *M. Tagawa & K. Iwatsuki T-4389* (L.2583137) • Doi Chiang Dao, alt. 1975 m, 10 November 1995, *J.F. Maxwell 95-1157* (Topotype, L.4214318).

##### Note.

*Hydrocotylechiangdaoensis* has been recorded in China, Myanmar, and Thailand. This species is well characterized by its palmate-dissected or irregular stipules, cymose terminal umbels, and papillose-setulous fruits. This species is restricted to limestone areas. *Hydrocotylechiangdaoensis* differs from *H.sibthorpioides* typically by its roots only growing at nodes of the basal stem and never elsewhere, with terminal cymose umbels and papillose-setulous fruits.

## Supplementary Material

XML Treatment for Hydrocotyle chiangdaoensis

## ﻿Additional information

### Author ORCIDs

Jun Wen https://orcid.org/0000-0002-1926-6517

Jun-Wen Zhu https://orcid.org/0009-0000-5554-445X

Ren-bin Zhu https://orcid.org/0000-0003-3925-1571

Chun-feng Song https://orcid.org/0000-0003-2077-0486

### Data availability

All of the data that support the findings of this study are available in the main text. Voucher specimens were identified by Jun Wen and deposited in NAS (Herbarium, Institute of Botany, Chinese Academy of Sciences, Jiangsu Province) with deposition numbers NAS00714697–NAS00714702. Raw reads of the chloroplast genome were uploaded to NCBI and placed under project PRJNA1221964 with accession number SRR32321003.
